# Antioxidant and Hepatoprotective Effect of *Penthorum chinense* Pursh Extract against *t*-BHP-Induced Liver Damage in L02 Cells

**DOI:** 10.3390/molecules20046443

**Published:** 2015-04-10

**Authors:** Yangyang Hu, Shengpeng Wang, Anqi Wang, Ligen Lin, Meiwan Chen, Yitao Wang

**Affiliations:** State Key Laboratory of Quality Research in Chinese Medicine, Institute of Chinese Medical Sciences, University of Macau, Macau 999078, China; E-Mails: umhuyangyang@163.com (Y.H.); sxwsp@163.com (S.W.); waqscu@hotmail.com (A.W.); ligenl@umac.mo (L.L.)

**Keywords:** *Penthorum chinense* Pursh, ROS, hepatoprotective effect, apoptosis

## Abstract

*Penthorum chinense* Pursh (*P. chinense*), a traditional Chinese medicine used by the Chinese Miao minority, has been used to treat liver diseases for a long time. However, the mechanism behind the liver protective effects of *P. chinense* remains unclear so far. The aim of the present study was to investigate the hepatoprotective effect of *P. chinense* and its possible mechanism(s). Immortalized normal human normal liver L02 cells were used to evaluate the protective effect of *P. chinense* aqueous extract against *tert*-butyl hydroperoxide (*t*-BHP)-induced liver cell damage. Treatment with *P. chinense* aqueous extract significantly protected L02 cells from *t*-BHP-induced cytotoxicity, prevented *t*-BHP-induced reactive oxygen species (ROS) generation and decreased the percentage of apoptosis by inhibiting the mitochondrial apoptotic pathway. This study demonstrates that *P. chinense* is a potential hepatoprotective agent in *t*-BHP-induced liver cell damage, which may benefit the further application of *P. chinense* in the clinic.

## 1. Introduction

*Penthorum chinense* Pursh (*P. chinense*), called Ganhuangcao in Chinese, belongs to the family Saxifragaceae and is widely distributed in eastern Asia, including China, Japan, Korea, and eastern Russia [1]. Daily consumption of *P. chinense* is recorded to be related with a decreased of liver diseases in Gulin country, Sichuan Province. Its favorable therapeutic effects in treating jaundice, cholecystitis, edema, traumatic injury and infectious hepatitis are also confirmed. Therefore, Gansu granules, a Chinese formulation, is prepared from *P. chinense* extracts and has been used in the clinic as a remedy for chronic hepatitis B and acute virus hepatitis for its action against the hepatitis B, C, and D viruses [2]. However, *P. chinense* has not been subjected to any detailed chemical constitution analysis and the mechanism of the liver protective effect of *P. chinense* remains unclear.

Production of reactive oxygen species (ROS) is implicated in normal aerobic cellular metabolism [3]. Generally, ROS production is counterbalanced by the antioxidant defense system to maintain an appropriate redox balance [4,5]. Oxidative stress, which is a physiological status whereby intracellular free radicals exceed the antioxidant abilities, has been recognized as a key factor in the pathogenesis of several chronic liver diseases, such as hepatitis, alcoholic and non-alcoholic fatty liver diseases [6,7]. The liver’s unique metabolic functions and relationships to the gastrointestinal tract make it vulnerable to the toxicity of drugs and xenobiotics [8,9]. Therefore, antioxidant therapy may be one of the strategies to correct the imbalance between oxidants and antioxidants in development of these liver diseases and prevent hepatocytes from excessive exposure to oxidative stress.

*tert*-Butyl hydroperoxide (*t*-BHP) is commonly used to induce oxidative stress *in vitro* and *in vivo*, and the induction of apoptosis in hepatocytes is a critical feature of *t*-BHP [10]. It can be metabolized to free radical intermediates, which further cause lipid peroxidation, GSH depletion and DNA damage [11]. Though *P. chinense* was reported to possess potent antioxidant capabilities [2,12,13], it still remains unclear whether the hepatoprotective effect of *P. chinense* could be verified by an ability to decrease *t*-BHP-induced oxidative stress and consequent apoptosis. Therefore, the objective of the present study was to investigate the potential protective effect and mechanism of *P. chinense* against oxidative stress induced by *t*-BHP in human liver L02 cells.

## 2. Results and Discussion

Oxidative stress is a situation whereby intracellular ROS/reactive nitrogen species (RNS) levels overwhelm the cellular antioxidant abilities and is reported to be associated with more than 100 diseases [3,8]. Basically, a normal ROS/RNS level and oxidative stress is essential for cell survival, while high levels of ROS/RNS or severe oxidative stress will inevitably weaken the cells’ self-repair capacity and lead to cell death thereof [3,14]. As the major organ responsible for the regulation of extensive physiological processes, the liver is a representative and target organ of the toxicity of drugs and xenobiotics due to its continuous exposure to these toxicants [9,15]. Most hepatotoxic chemicals can increase the production of free radicals that cause oxidative stress, which is considered as one of the important mechanisms that mediate liver toxicity [16]. *P. chinense*, a Miao ethnic medicine, has been applied for treatment of liver diseases for many years. We postulated that *P. chinense* could protect the liver from cell injury induced by *t*-BHP, a well-known ROS inducer, based on previous studies indicating that *P. chinense* possesses ROS scavenging activity [2,12].

### 2.1. Chemical Characteristics of P. chinense Extract

**Figure 1:**
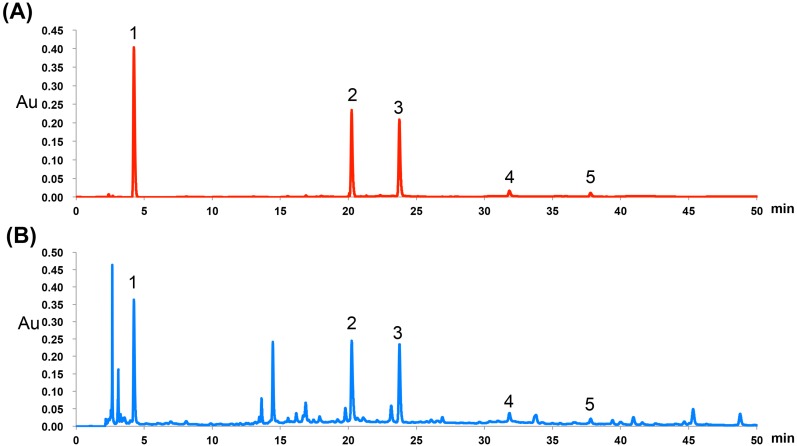
Representative HPLC-UV chromatograms of mixed standards (**A**) and *P. chinense* extract (**B**). Gallic acid (1), isoquercitrin (2), quercitrin (3), quercetin (4) and kaemferol (5).

Previous studies showed that *P. chinense* is rich in polyphenols, which possess strong antioxidant activities [12,17]. In order to investigate the chemical characteristics of *P. chinense* extract used in present study, HPLC analysis of *P. chinense* extract was carried out and confirmed the dominant presence of polyphenols in the *P. chinense* extract as anticipated (Figure 1). The polyphenols were identified by comparison of the retention times with authentic mixed polyphenol standards. Five peaks were identified as gallic acid, isoquercitrin, quercitrin, quercetin and kaempferol, which is consistent with previous reports [17]. The contents of the five compounds were quantified using corresponding chemical standards. Specifically, the contents of gallic acid, isoquercitrin, quercitrin, quercetin and kaempferol in *P. chinense* extract were 5.50, 14.1, 10.4, 0.8 and 0.1 mg/g, respectively.

### 2.2. Protective Effect of P. chinense Extract on t-BHP-Induced Cytotoxicity in L02 Cells

**Figure 2:**
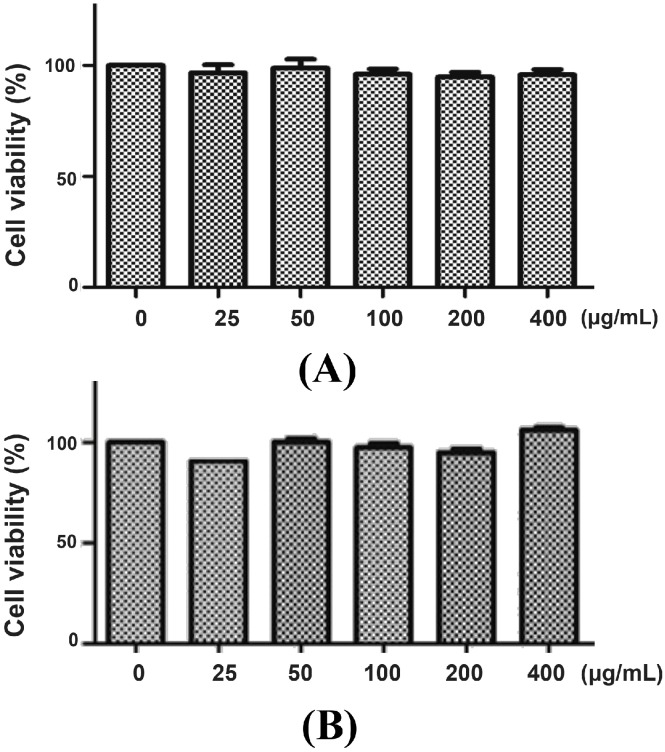
Cytotoxicity of *P. chinense* extract on L02 cells. L02 cells were treated with different concentrations of *P. chinense* extract for 12 h (**A**) and 24 h (**B**), then cell viability were assessed by MTT assay. Data are expressed as means ± SEM of at least three independent experiments.

**Figure 3:**
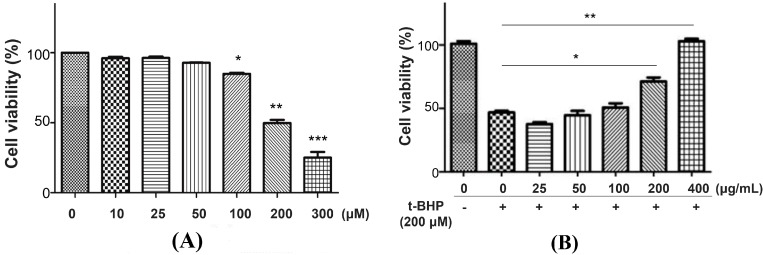
The protective effects of *P. chinense* extract on *t*-BHP-induced cytotoxicity in L02 cells. (**A**) L02 cells were treated with different concentrations of *t*-BHP for 6 h. * *p* < 0.05, ** *p* < 0.01 and *** *p* < 0.001 as compared to untreated control cells. (**B**) L02 cells were pretreated with different concentrations of *P. chinense* extract for 12 h, followed by treatment with 200 µM *t*-BHP for another 6 h. Cell viability was assessed by MTT assay. Data are expressed as means ± SEM of at least three independent experiments. Con indicates control. * *p* < 0.05 and ** *p* < 0.01.

We first determined the cytotoxicity of *P. chinense* extract in L02 cells. After 12 h (Figure 2A) and 24 h (Figure 2B) treatment, *P. chinense* extract showed negligible toxic effect on L02 cells even at high concentration (400 µg/mL). Then, *t*-BHP, a potent pro-oxidant, was used to induce oxidative damage in L02 cells [18]. As shown in Figure 3A, *t*-BHP (25 to 400 µM) treatment for 6 h evoked the decrease of L02 cell viability in a concentration-dependent manner, with an IC_50_ value of 205.8 µM. A 6 h treatment of 200 µM *t*-BHP, which induced almost 50% decrease of cell viability, was used to evaluate the potential hepatoprotective ability of *P. chinense* extract in the subsequent studies.

Pretreatment of L02 cells with 25, 50 and 100 µg/mL *P. chinense* extract for 12 h showed weak protective effect on *t*-BHP-induced cytotoxicity, while 200 µg/mL of *P. chinense* extract significantly reduced cell damage. A higher dose of *P. chinense* extract (400 µg/mL) further reduced cell damage to values similar to those of control cells (Figure 3B), indicating the strong protective activity of *P. chinense* extract against *t*-BHP-induced cytotoxicity in L02 cells.

### 2.3. P. chinense Extract Decreased ROS Generation in L02 Cells

**Figure 4:**
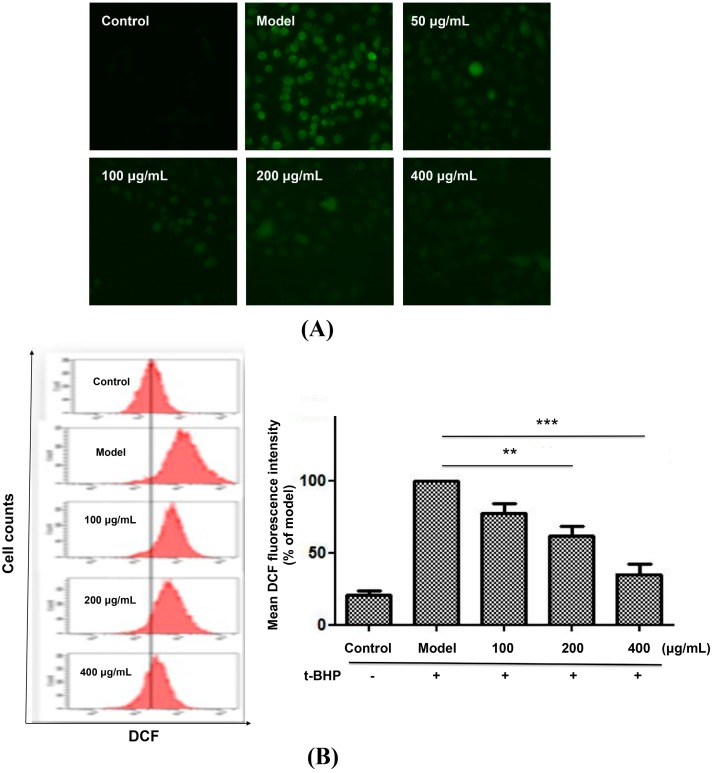
*P. chinense* extract attenuated *t*-BHP-induced ROS generation in L02 cells. L02 cells were pretreated with different concentrations of *P. chinense* extract for 12 h, followed by exposition to *t*-BHP for 30 min, and then incubated with 1 µM (CM-H_2_DCF) for another 30 min. The intracellular ROS levels were measured by In Cell Analyzer 2000 (**A**) and flow cytometry (**B**). Data are expressed as means ± SEM of at least three independent experiments. ** *p* < 0.01 and *** *p* < 0.001. Magnification 200×.

It is well known that ROS play an important role in cell apoptosis [19]. Measurement of intracellular ROS levels is a common indication of the oxidative damage to living cells. To investigate whether the protective effect of *P. chinense* extract on *t*-BHP-induced cytotoxicity was attributed to the intracellular ROS levels, we further determined the intracellular ROS levels using a ROS-specific dye (CM-H_2_DCFDA). The regulation of ROS generation was evaluated in L02 cells treated by *t*-BHP with or without *P. chinense* extract pretreatment. As shown in Figure 4A, *t*-BHP treatment significantly increased the intracellular ROS level compared with that of untreated controls, while pretreated with *P. chinense* extract greatly decreased ROS production to those of untreated cells.

Flow cytometry studies further indicated that, compared with model group, pretreated with 100, 200 and 400 µg/mL *P. chinense* extract obviously decreased the ROS levels to 77.48%, 61.90% and 35.25% of the model cells (Figure 4B), respectively. These results clearly showed that *P. chinense* extract strongly inhibits the generation of ROS induced by *t*-BHP, thus preventing or delaying conditions that favor oxidative stress in liver cells.

### 2.4. P. chinense Extract Attenuated t-BHP-Induced Apoptosis

**Figure 5:**
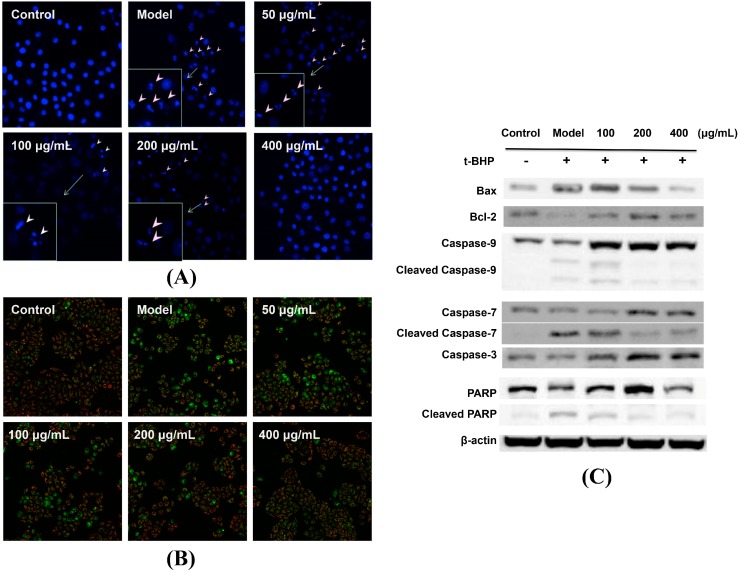
*P. chinense* extract attenuated *t*-BHP-induced apoptosis by inhibiting mitochondrial apoptotic pathway in L02 cells. (**A**) L02 cells were pretreated with different concentrations of *P. chinense* extract for 12 h, followed by exposition to *t*-BHP for 30 min, and then incubated with JC-1 dye for another 30 min. The cell MMP was measured using In Cell Analyzer 2000. (**B**) L02 cells were treated as described above and cell nuclei were stained with Hoechst 33342 and imaged using an In Cell Analyzer 2000. (**C**) L02 cells were treated as described above and were harvested for western blotting analysis using indicated antibodies. Similar results were obtained from two or three independent experiments. Magnification 200×.

The induction of apoptosis in hepatocytes by *t*-BHP is a critical feature of oxidative agents [20]. We further evaluate the effect of *P. chinense* extract on the apoptosis of L02 cells induced by *t*-BHP. The depolarization of MMP is an essential event for detecting initial apoptosis [21]. In this study, we used JC-1 as the fluorescent dye and estimated the change of MMP through the intensity weaken of JC-1 aggregates, which may reduce as MMP decreases. As shown in Figure 5A, *t*-BHP treatment greatly increased the green florescence, suggesting the significant decrease of MMP. However, cells pretreated with *P. chinense* extract inhibited the decrease of MMP by a concentration-dependent manner (Figure 5A). Hochest 33342 staining also showed that L02 cells pretreated with *P. chinense* extract reduced condensed, fragmented or degraded cell nuclei as compared to *t*-BHP-treated group (Figure 5B).

In order to investigate the protective mechanisms of *P. chinense* extract, the expression of several apoptosis-related proteins were determined by western blotting in L02 cells. Caspases as potent executors of apoptosis exist as zymogens in normal cells and undergo proteolytic processing via several pathways [22]. PARP is involved in many cellular processes including DNA repair and cell apoptosis [23], and cleaved PARP is recognized as a marker of apoptosis. Generally, apoptosis pathways can be divided into death receptors pathway and mitochondria pathway. ROS are predominantly produced in mitochondria. It has been reported that the mitochondria-dependent apoptotic pathway is involved in *t*-BHP-induced cytotoxicity, in which the Bcl-2 family proteins play a key role [24]. The anti-apoptotic Bcl-2 bind to the outer membrane of the mitochondria can stabilize the membrane permeability and prevent the release of cytochrome c [25], while the pro-apoptotic members like Bax are responsible for permeabilizing membrane under cellular stress [26,27]. *t*-BHP treatment increased the expression of Bax and cleaved products of caspase-9, caspase-7 and PARP, while decreased the level of Bcl-2 (Figure 5C). We found that *P. chinense* extract pretreatment could counteract *t*-BHP-induced upregulation of Bax and cleaved products of caspase-9, caspase-7 and PARP, as well as downregulated Bcl-2 expression (Figure 5C), suggesting that *P. chinense* extract may attenuate *t*-BHP-induced apoptosis by inhibiting mitochondrial apoptotic pathway in L02 cells.

## 3. Experimental Section

### 3.1. Chemicals and Reagents

Phosphate-buffered saline (PBS) powder, RPMI 1640 medium, penicillin-streptomycin (PS), 0.25% (w/v) trypsin/1 mM EDTA, fetal bovine serum (FBS), CM-H_2_DCF-DA and JC-1 dyes were purchased from Life Technologies (Grand Island, NY, USA). *tert-*Butyl hydroperoxide (*t*-BHP) and 3-[4,5-dimethylthiazol-2-yl]-2,5-diphenyl-2*H*-tetrazolium bromide (MTT) were purchased from Sigma-Aldrich (St. Louis, MO, USA). All primary antibodies and the secondary antibodies were purchased from Cell Signaling Technology (Danvers, MA, USA). The chemical standards including gallic acid, isoquercitrin, quercitrin, quercetin and kaemferol were obtained from Dalian Meilun Biology Technology Co., Ltd. (Dalian, Liaoning, China).

### 3.2. Preparation of P. chinense Extract

The dried *P. chinense*, obtained from Luzhou city (Sichuan, China), was provided by Neautus Traditional Chinese Medicine Co. (Sichuan, China). Briefly, the sliced crude herb materials were extracted for three times by decoction, 1 h each time. After filtration, the solution was evaporated under reduced pressure to obtain an extract at a yield of 11.6% (w/w) and stored at −20 °C for further use.

### 3.3. HPLC Analysis of P. chinense Extract

The *P. chinense* extract (100 mg) was suspended in distilled water (100 mL) and sonicated for 30 min. After centrifugation at 3000 rpm for 10 min, the supernatant was filtered through a 0.45 µm membrane. The subsequent filtrate was then analyzed using an e2695 HPLC system (Waters, Milford, MA, USA) equipped with a UV detector. HPLC was achieved using a Zorbax SB-C18 column (250 mm × 4.6 mm, 5 µm; Agilent, Santa Clara, CA, USA) with a gradient elution program at a flow rate of 1.0 mL/min. The mobile phase was composed of 0.01% aqueous trifluoroacetic acid (A) and acetonitrile (B), and the following gradient was applied: 0–6 min, 95% A; 6–12 min, 95%–85% A; 12–55 min, 85%–50% A. The column temperature was maintained at 35 °C, the detection wavelength was set at 254 nm, and the sample injection volume was 20 µL. Representative chromatograms of the *P. chinense* extract were analyzed using a Waters Empower system.

### 3.4. Cells and Cell Culture

The immortalized normal human liver cell line L02 was obtained from Shanghai Institute of Biochemistry and Cell Biology (Shanghai, China). Cells were cultured in RPMI 1640 medium supplemented with 10% FBS, 100 U/mL penicillin and 100 mg/mL streptomycin at 37 °C in a 5% CO_2_ humidified environment.

### 3.5. Cell Viability Assay

The cell viability was estimated by MTT assay as described previously [28]. L02 cells were seeded in 96-well plates at a density of 5 × 10^3^ cells/well. After reaching approximately 70%–80% confluence, cells were treated with indicated drugs. Cell viability was determined by being incubated with medium containing MTT (1 mg/mL) for 4 h, followed by dissolving the formazan crystals with DMSO. The absorbance at 570 nm was determined by a microplate reader (SpectraMax M5, Molecular Devices, CA, USA) and presented as relative cell viability. The results were analyzed based on at least three independent experiments.

### 3.6. Measurement of Reactive Oxygen Species

A fluorescent probe, 5-(and-6)-chloromethyl-2',7'-dichlorodihydrofluorescein diacetate (CM-H_2_DCFDA, Life Technologies, East Rutherford, NJ, USA), was used for determination of intracellular ROS levels. Cells were pretreated with different concentrations of *P. chinense* extract for 12 h and then refreshed the medium and incubated with *t*-BHP for another 30 min. After staining with CM-H_2_DCF-DA (1 µM) for 15 min, cells were gently washed with PBS and harvested for analysis by flow cytometry (Becton Dickinson, Franklin Lakes, NJ, USA).

### 3.7. Mitochondrial Membrane Potential (MMP) Assay

The mitochondrial membrane potential was monitored using fluorescent probe JC-1 staining. Briefly, L02 cells were pretreated with different concentrations of *P. chinense* extract for 12 h, followed by refreshing the medium and treating with *t*-BHP for 30 min. The cells were stained with JC-1 (1 μg/mL) for another 10 min. The fluorescence of JC-1 was observed with In Cell Analyzer 2000 (GE Healthcare Life Sciences, Logan, UT, USA).

### 3.8. Hochest 33342 Staining

Hochest 33342 staining was performed as previously reported [28]. In brief, L02 cells were treated with different concentrations of *P. chinense* extract for 12 h and then refreshed the medium and incubated with *t*-BHP for another 6 h. The cells were fixed with 4% paraformaldehyde for 30 min at room temperature and stained with Hochest 33342 (1 µg/mL) for 10 min. The apoptotic morphology of cell nuclei was observed using In Cell Analyzer 2000 (GE Healthcare Life Sciences, Logan, UT, USA).

### 3.9. Western Blot

Cells were treated as described above, and western blot analyses were conducted as previously described [29]. Briefly, the total protein was extracted using RIPA lysis buffer containing 1% phenylmethanesulfonylfluoride (PMSF) and 1% protease inhibitor cocktail. Protein concentrations were quantified with a BCA protein assay kit (Life Technologies, Grand Island, NY, USA) and equivalent amounts of proteins from each group were separated by SDS-PAGE, followed by transferring onto PVDF membranes. After blocking with 5% nonfat milk at room temperature for 1 h, the membrane was incubated with specific primary antibodies and corresponding secondary antibodies. Specific protein bands were visualized using an ECL advanced western blotting blot kit (GE Healthcare Life Sciences). Band detection was calculated with the Quantity one software (Bio-Rad, Hercules, CA, USA).

### 3.10. Data Analysis

All the data expressed in the mean of three separately performed experiments and were presented as mean ± SEM. Statistical analysis was carried out using GraphPad Prism 5 software (GraphPad Software, San Diego, CA, USA). One-way ANOVA was used for statistical comparison, and *p*-values less than 0.05 were considered statistically significant.

## 4. Conclusions

Taken together, our results indicate that the aqueous extract of *P. chinense*, which does not show significant toxicity up to 400 μg/mL, could serve as a candidate with strong hepatoprotective effects in *t*-BHP-induced liver cell damage, by mediating ROS scavenging and inhibition of the mitochondrial apoptotic pathway. These findings may benefit the further application of *P. chinense* in the clinic. However, although oxidative stress and ROS were implicated in the hepatoprotective effect *P. chinense* in this study, future metabolomics and/or transcriptomics studies are still required to investigate the specific metabolic or signaling transduction pathways involved in this process.
